# Dentoskeletal Class II Malocclusion: Maxillary Molar Distalization with No-Compliance Fixed Orthodontic Equipment

**DOI:** 10.3390/dj8010026

**Published:** 2020-03-18

**Authors:** Vincenzo Quinzi, Enrico Marchetti, Luigi Guerriero, Floriana Bosco, Giuseppe Marzo, Stefano Mummolo

**Affiliations:** 1 Department of Life, Health and Environmental Sciences, University of L’Aquila, Piazzale Salvatore Tommasi 1, L’Aquila 67100 Coppito, Italy; vincenzo.quinzi@univaq.it (V.Q.); enrico.marchetti@univaq.it (E.M.); luigi.guerriero@univaq.it (L.G.); giuseppe.marzo@univaq.it (G.M.); 2 Private practice in 20121 Milan, Italy; boscofloriana@gmail.com

**Keywords:** no-compliance orthodontic appliance, class II malocclusion, fied orthodontic appliance, systematic review

## Abstract

Dentoskeletal class II malocclusion due to a protruded upper dental arch is a major reason for an orthodontic treatment. In these cases, the correction of class II can be hindered by molar distalization, obtained with ‘no-compliance therapy’ that involves the use of appliances which minimize the need for such co-operation and attempt to maximize the predictability of results. The aim of this review was to outline the effectiveness of no-compliance fixed orthodontic devices in the molar distalization. After selection according to the inclusion/exclusion criteria, 16 articles from 2000 to 2019 were qualified for the final analysis. The literature shows various no-compliance fixed devices whose effect is to distalize the maxillary molars. The present revision allows to conclude that there is a need to increase the number of studies, especially with regard to the most recently introduced devices in the literature. The analysed studies allow to hypothesize that these appliances act with a minimal variability of molar distalization and disto-inclination among them, although different effects among the appliances can be observed as regards to the anchorage.

## 1. Introduction

Modern orthodontics is active in continuous research of non-invasive treatments, respecting human dental and biological characteristics [1], trying to reserve extraction methods in extreme cases [2], when the absence of space prevents the alignment of teeth. Teeth extractions may be reduced thanks to the use of equipment that increases the transverse and sagittal dimensions of the dental arches. Patients with class II malocclusion represent about 35% of cases in the American and European population [3]. Class II malocclusions have also been correlated to temporomandibular joint dysfunctions [4,5] and wrong posture in children [6]. 

Class II malocclusion is given by a correlation of a sagittal relationship with mandibular retrognathia and/or maxillary protrusion of varying degrees and entities [7].

There are various types of equipment to treat this malocclusion, from functional therapy (applied in growing patients) to fixed mechanicals, correlated not only to the degree of musculoskeletal maturation of the patient [8], to the oral functional habits as oral breathing [9], but also to the psycho-social state of the patient and to their compliance [10,11,12].

Removable functional orthopaedic appliances are reserved for Class II treatment of growing patients with normal position and growth of the maxilla, and with mandibular retrusion, to be used during their circumpubertal growing stage [13,14,15] and who, in any case, show a good degree of therapy acceptance and therefore, collaboration.

The biological mechanism of these appliances is based on the anatomy of the temporomandibular joint (TMJ) and its development during the growing period. During that period, the TMJ area has been observed to be a reactive growth site. In particular, during the period of mixed dentition, TMJ is affected by a considerable amount of growth and adaptation, which can correct the jaws relationships thanks to an interceptive functional orthodontic treatment [16,17]. 

When the class II malocclusion is due to mandibular skeletal retrusion, the literature agrees that it is appropriate to address the problem with a treatment that stimulates mandibular growth. There is a good range of functional devices to stimulate the “forward postural growth” of the jaw, both removable (the Fraenkel II, the Bionator, and the Twin Block appliances are among the most used) and fixed devices (the Forsus Fatigue Resistant appliance, the Herbst appliance, the Jusper Jumper appliance, and the Carriere Motion 3D appliance are among the most used). No-compliant fixed appliances are anchored to distal parties of maxillary arch and to mesial parties of mandibular arch [10,11,12] and can be also anchored to miniscrews to minimize their effect on anchoring teeth [18]. Cozza and Baccetti reviewed 704 articles, published in international journals from January 1966 to January 2005 [15]. That study found that the average functional coefficient for mandibular orthopaedic growth was 0.16 mm per month. From that study, it was evidently deduced that none of the analysed devices causes clinically significant mandibular growth change, because all the total increases of mandibular length are generally less than 2 mm during functional therapy. However, that study evidently clarified that the jaw progress with the functional treatment is a consequence of the biological reaction of the condylar cartilage, particularly reactive in the period of skeletal maturation (at the turn of the puberty peak of growth). During that period of skeletal maturation, the “forward postural mandibular growth” is the most effective strategy to correct class II malocclusion (with an average value of 5.1 mm). The interceptive treatment is based on a change in the mandibular postural position [19] that persists after the active treatment [20]. Forward mandibular positions have also been associated to a general improving of masticatory muscular function [21], and neck muscles [22], compared to a growth stimulated in the pre-pubescent or post-pubescent period. On the basis of these data, the diffusion of “forward postural mandibular growth” treatments for class II malocclusion can be confirmed due to retro-positioned mandible in growing subjects during the phase of skeletal maturation [23]. 

For class II patients with a normal position of the mandible and maxillary dento-skeletal class II malocclusion, even during the growing period, the molar distalization is a diffused strategy of treatment, to achieve a correction of the molar relationship. Molar distalization is also required in patients after the circumpubertal stage of growing, with class II malocclusions characterized by dento-skeletal protrusion of the upper dental arch and normal position of the lower arch. Molar distalization can be achieved with fixed no-compliant devices, that are often the same devices used for protruded maxillary arches. The Herbst appliance is a fixed appliance that allows the forward repositioning of the jaw, keeping it always present both with the mouth closed and open, allowing the correction of the discrepancy. The Distal Jet, the First Class, the Pendulum and the Carriere 3D motion are fixed only on the upper dental arch. The Jusper Jumper and the Forsus appliances are fixed to the distal part of the upper arch, and the mesial part of the lower arch. The Distal Jet and the First Class have the force anchored to a palatal button and to anterior teeth. The Distal Jet is based on two Ni-Ti coil springs that are positioned in the palatal side of the teeth, to be near the resistance centre of the teeth. 

The First class has two coil springs in stainless steel that are positioned on the vestibular side of the molars, between the upper first molars and the first upper bicuspids. The Carries 3D motion is made by a bar that is anchored to the vestibular side of the upper first molar and the canine. The Pendulum appliance is anchored to the upper dental arch, and its distalization force is due to a wire toe. All these appliances can be used with the multibrackets device. Among distalizating appliances, there is also the EOT (Extra Oral Traction), a removable appliance, for which the therapeutic efficacy is directly related to the patient’s degree of collaboration [24], both with the appliance management and the oral hygiene [25].

The aim of this review was to outline the effectiveness of fixed no-compliance orthodontic devices to obtain a molar distalization, and evaluate other related teeth movements. 

## 2. Materials and Methods 

In the present literature review, an electronic search was carried out using EMBASE; Web of Science and Scopus. Moreover, Cochrane database and Google scholar were reviewed.

The P.I.C.O. question was to compare the “molar distalization” (primary outcome) measured in degrees or millimetres, and other teeth movements observed during the molar distalization (secondary outcome), obtained with various fixed no-compliance orthodontic devices (intervention), in a population of children/adolescents/adult subjects, who needed the upper first molar distalization for the correction of a class II relationship. 

To identify records, the research strategy initially included 3 sets of mesh terms: records related to health conditions (the mesh term “class II malocclusion”), records relating to the intervention to be evaluated (the mesh term “no-compliance fixed therapy”), and records that identify the design of the study to be included. Since a pilot series of the strategy concerning the study design did not produce articles from any database, the present search was limited to the first 2 terms.

No language restriction was applied, nor any filter during the research.

The review was carried out following the P.R.I.S.M.A. declaration (Preferred Reporting Items for Systematic Review and Meta-analysis). 

Inclusion criteria were

- Articles published between 2000 and 2019;
- Randomized controlled clinical trials were always included, because they probably contain evidence of acceptable quality;
- Prospective and retrospective studies were accepted only if they included more than 20 treated subjects per intervention group; and with comparisons with an untreated class II malocclusion group, and/or, alternatively, a treated class II malocclusion group;
- Case-reports were also evaluated, and included only if they showed the two basic terms.

Exclusion criteria were those reports that did not include data on the stability of treatment, for at least one year, regarding skeletal changes and/or soft tissue changes and/or dental and occlusal changes (preferably evaluated with an occlusal index); and also the gum health, the state of the temporomandibular joint or relative muscular activity; and/or the quality of life. 

**Figure 1:**
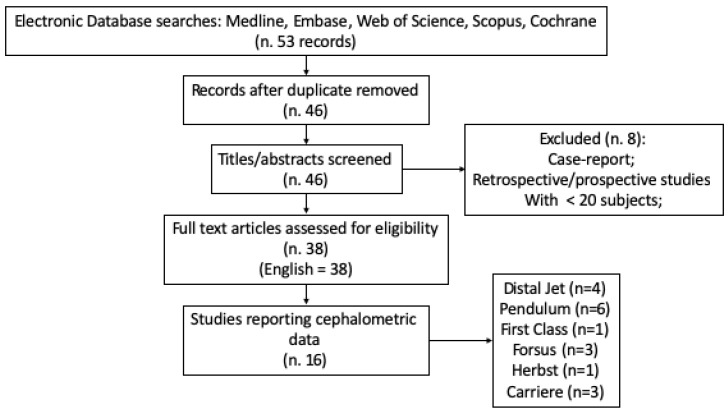
Flow chart of the research strategy.

The flow chart of the study is reported in Figure 1.

The selection of articles, the decisions on their admissibility, the classifications of studies, and data extraction were carried out independently and in duplicate by 2 operators, without blinding on the types of devices, or the obtained results. After the inclusion, the following information were recorded for each admitted study on a data collection form: the initials of the reviewer, the authors, the year of publication, the setting of the study, age and sex of the subjects, the design of the study, the definition of the criteria for malocclusion, the calculation sample size, the type and duration of treatment, the dropouts, the type of storage, the outcome measures, the assessment methods, the study of errors and, finally, the results of the study.

## 3. Results

A total group of 38 articles met inclusion criteria, and were recruited for the critical exam. 

Among them, 16 articles reported cephalometric data [10,12,26,27,28,29,30,31,32,33,34,35,36,37,38,39].

The flow chart of the research strategy is represented in Figure 1.

The quality analysis of the included studies gave acceptable results. All the included studies described a method error analysis to evaluate the intra-examiner method error, and in some cases, the inter-examiners method error that resulted was always acceptable, with a minimization of the bias associated to the calculation of values. Analyses of bias showed that none of the studies used random sequence generation, blinded participants and personnel or used blinding of outcome assessments.

As the primary outcome of the present review was to compare the effectiveness of fixed no-compliance orthodontic devices to obtain the molar distalization, this effect, together with other teeth movements (considered as secondary outcome) are presented in the Table 1 for a total number of 2240 of patients (1101 males and 1139 females), with start age of therapy of 10.9 years +/− 2.9 months. 

The upper first molar (U6) distalization was found with an average value of 3.14 mm, with a maximum value for the Pendulum appliance (5.4 mm) and a minimum value for the Forsus appliance and the Herbst appliances (1.45 mm). 

The disto-inclination of U6 was on average of 8.34 degrees, with a maximum value for the Forsus appliance (23.92 degrees), and a minimum value for the Pendulum K appliance (3.36 degrees).

About the anchorage to the other teeth (secondary outcome), the following data were recorded from the considered sample.

The mesialization of the lower first incisive (L1) showed an average value of 2.16 mm, with a maximum value for the Forsus appliance and the Carriere appliance (2.3 mm), and a minimum value for the Herbst appliance (1.85 mm). The Forsus appliance and the Herbst appliances also determined a vestibular-inclination of L1 (of 9.29 degrees, and 5.95 degrees, respectively).

The mesialization of the lower first molar (L6) showed an average value of 2.1 mm, with a maximum value for the Carriere appliance (2.7 mm) and a minimum value for the Herbst appliance (1.5 mm), with a mesial-version of 3.03 degrees at mean, with a maximum for the Carriere appliance (4.15 degrees) and a minimum value for the Herbst appliance (1.7 degrees).

## 4. Discussion

In order to obtain a molar distalization, the orthodontic technology, in recent years, has proposed various no-compliance fixed orthodontic devices, whose effect is to distalize the maxillary molars. Appliances such as the Pendulum, the Distal Jet, the First class, the Carriere Motion are among the most used, with a minimal variability of molar distalization and disto-inclination, as deduced from the present literature review (Table 1). The present data confirm the validity of the approach based on these appliances.

Effects of different entity were observed with regards to the anchorage of the other teeth.

Some differences were observed in the anchorage, among the devices that adopt a resin palatal button as an anchor, and the devices that do not include it (Table 1). The different design of the devices could influence their effect on the anchoring teeth. Where the palatal button is present, for example, the anchoring of the upper anterior teeth can be improved. As seen in Table 1, the Distal Jet, the Pendulum, and the First class appliances showed U5 mesialization, and U1 vestibular version. While, the Forsus, the Herbst and the Carriere appliances showed U1 lingual version, and, in the lower dental arch, L6 mesialization, and L1 vestibular version. Analogue differences were also observed for the angular movements (Table 1)

The present data can be compared with other studies that are based on an adult sample.

In a sample of 33 adult patients, Cozzani et al., without considering the adopted therapy, reported that a U6 distalization of 2.9 +/− 0.6 mm at mean, lower than the present data (3.14 mm at mean), allows to solve the class II molar relationship in about 64% of patients [40]. 

A similar finding was also reported for the 21 adult patients treated by Ravera and Castroflorio with clear aligners, where the values of U6 distalization of 2.25 mm at mean was reported, without significant any extrusion of the same tooth [36] and more recently, from Caruso et al. [41]

For some of the considered appliances, there are also data concerning their acceptance by patients, as for example for the Forsus appliance [38], and for the Carrier 3D Motion appliance [42]. The following considerations are generally derived from these studies: the great part of patients report a difficulty to eat soon after positioning the fixed device, improving with time. Sleep and personal appearance were sometime negative with each of the considered fixed devices. Regarding the side effects (toothache, wide opening difficulties, aching jaws, difficulty in keeping the device clean, soreness on the lip/cheek by rubbing), no significant difference was reported among the various devices, except for the pain of rubbing and drooling, worse with the Forsus appliance. The great part of subjects reported that the appliances had no effect on school work and their relationships with family and friends. Both musical activities and sports activities were not influenced by the appliances. The breakdown of the device, which required an extra visit from the orthodontist, occurred more frequently for the Forsus appliance. 

On the basis of the articles analysed in the present review, it can be confirmed that the trend of the past twenty years was to use no-compliance fixed appliances for molar distalization in cases of non-extractive treatments of Class II malocclusion after the pubertal stage. 

## 5. Conclusions

The literature from the past twenty years shows various no-compliance fixed devices, whose effect is to distalize the upper maxillary molars. From the data in the literature, it can be deduced that there is a fair number of devices available to clinicians. However, the low number of studies for some more recent devices does not allow an adequate statistical comparison among the devices. The present revision allows to hypothize that these appliances act with a minimal variability of molar distalization and disto-inclination among them, although different effects among the appliances can be observed as regards to the anchorage. However, there is a need to increase the number of studies especially with regard to the most recently introduced devices in the literature.

## Figures and Tables

**Table 1:** Results of the various appliances.

	Distal Jet	Pendulum	Pendulum K	Pendulum BaPa	First Class (Leone)	Forsus Appliance	Herbst Appliance	Carriere Appliance
U6 distalization (mm)	1.9/3.7 [26,33,34,35]	4 [26,28,29,30,35] (on average)	4 [26,28,29,31] (on average)	5.4 [26,28,29,31] (on average)	4 [40] (on average)	1.45 [10,38,40] (on average)	0.2/2.7 [27]	1.6/2.5 [37,39,42]
U6 tip distal (degree)	3.1/7.3 [26,33,34,35]	6 [26,28,29,30,35] (on average)	−0.83/+5.89 [26,28,29,31]	-	4.6 [40] (on average)	23.92 [10,38,40] (on average)	5.3/7.9 [27]	0.2/3.7 [37,39,42]
L6 Mesialization (mm)	-	-	-	-	-	1.7/2.4 [10,38,40]	1.3/1.7 [27]	0.9/3.1 [37,39,42]
L6 mesioinclination (degree)	-	-	-	-	-	1.9/4.6 [10,38,40]	1.2/2.2 [27]	2.2/6.1 [37,39,42]
U1 lingual version (mm)	-		-	-	-	3.16 [10,38,40] (on average)	1.1/2.3 [27]	0.6/2.4 [37,39,42]
U1 lingual inclination (degree)	-	-	-	-	-	8.94 [10,38,40] (on average)	7.5/9.6 [27]	5.4/6.8 [37,39,42]
L1 vestibule version (mm)	-	-	-	-	-	2.34 [10,38,40] (on average)	1.3/2.4 [27]	1.9/2.7 [37,39,42]
L1 vestibular inclination (degree)	-	-	-	-	-	9.29 [10,38,40] (on average)	3.6/8.3 [27]	6.3 [37,39,42] (on average)
U5 mesialization (mm)	0.4/3 [26,33,34,35]	1 [26,28,29,30,35] (on average)	1 [26,28,29,31] (on average)	-	1.7 [40] (on average)	-	-	-
U6 mesial space (mm)	2.3/6.7 [26,33,34,35]	5 [26,28,29,30,35] (on average)	5 [26,28,29,31] (on average)	5.4 [26,28,29,31] (on average)	5.7 [40] (on average)	-	-	-
U1 vestibular version (mm)	1.7 [26,33,34,35] (on average)	1.6 [26,28,29,30,35] (on average)	0.8 [26,28,29,31] (on average)	-	0.9 [40] (on average)	-	-	-
U5 distalization (mm)	-	-	-	3.8 [26,28,29,31] (on average)	-	-	-	-
U1 vestibular inclination (degree)	-	-	-	-	2.6 [40] (on average)	-	-	-
U1 anchor loss (mm)	-	-	-	0 [26,28,29,31] (on average)	-	-	-	-
U6 = upper first molarL 6 = lower first molar U1 = upper first incisor L1 = lower first incisor U5 = upper second bicuspid								

## References

[1] Mummolo S., Nota A., De Felice M.E., Marcattili D., Tecco S., Marzo G. (2018). Periodontal status of buccally and palatally impacted maxillary canines after surgical-orthodontic treatment with open technique. J. Oral Sci..

[2] Dinoi M.T., Mummolo S., Monaco A., Marchetti E., Campanella V., Marzo G. (2019). Correction to: Orthodontic treatment of the transposition of a maxillary canine and a first premolar: A case report. J. Med. Case Rep..

[3] Proffit W.R., Fields H.W., Moray L.J. (1998). Prevalence of malocclusion and orthodontic treatment need in the United States: Estimates from the NHANES III survey. Int. J. Adult Orthodon. Orthognath. Surg..

[4] Tecco S., Festa F., Salini V., Epifania E., D’Attilio M. (2004). Treatment of Joint Pain and Joint Noises Associated with a Recent TMJ Internal Derangement: A Comparison of an Anterior Repositioning Splint, a Full-Arch Maxillary Stabilization Splint, and an Untreated Control Group. Cranio.

[5] Silvestrini-Biavati A., Migliorati M., Demarziani E., Tecco S., Silvestrini-Biavati P., Polimeni A., Saccucci M. (2013). Clinical association between teeth malocclusions, wrong posture and ocular convergence disorders: An epidemiological investigation on primary school children. BMC Pediatr..

[6] Mummolo S., Tieri M., Tecco S., Mattei A., Albani F., Giuca M.R., Marzo G. (2014). Clinical evaluation of salivary indices and levels of Streptococcus mutans and Lactobacillus in patients treated with Occlus-o-Guide. Eur. J. Paediatr. Dent..

[7] McNamara J.A. (1981). Components of class II malocclusion in children 8–10 years of age. Angle Orthod..

[8] Alarashi M., Franchi L., Marinelli A., Defraia E. (2003). Morphometric analysis of the transverse dentoskeletal features of class II malocclusion in the mixed dentition. Angle Orthod..

[9] Mummolo S., Nota A., Caruso S., Quinzi V., Marchetti E., Marzo G. (2018). Salivary Markers and Microbial Flora in Mouth Breathing Late Adolescents. Biomed Res. Int..

[10] Franchi L., Alvetro L., Giuntini V., Masucci C., Defraia E., Baccetti T. (2011). Effectiveness of comprehensive fixed appliance treatment used with the Forsus Fatigue Resistant Device in Class II patients. Angle Orthod..

[11] Keim R.G., Gottlieb E.L., Nelson A.H., Vogels D.S. (2008). 2008 JCO study of orthodontic diagnosis and treatment procedures, part 1: Results and trends. J. Clin. Orthod..

[12] Dalci O., Altug A.T., Memikoglu U.T. (2014). Treatment effects of a twin-force bite corrector versus an activator in comparison with an untreated Class II sample: A preliminary report. Aust. Orthod. J..

[13] D’Attilio M., Tecco S., Filippi M.R., Delli Carri D., Festa F. (2001). Activation of mandibular growth with the Fräenkel II device: Assessment of effects on the vertical plane | Attivazione della crescita mandibolare mediate il Fräenkel II: Valutazione degli effetti sul piano verticale. Minerva Stomatol..

[14] Koretsi V., Zymperdikas V.F., Papageorgiou S.N., Papadopoulos M.A. (2015). Treatment effects of removable functional appliances in patients with Class II malocclusion: A systematic review and meta-analysis. Eur. J. Orthod..

[15] Cozza P., Baccetti T., Franchi L., De Toffol L., McNamara J.A. (2006). Mandibular changes produced by functional appliances in Class II malocclusion: A systematic review. Am. J. Orthod. Dentofacial Orthop..

[16] Tecco S., Baldini A., Nakaš E., Primozic J. (2017). Interceptive Orthodontics and Temporomandibular Joint Adaptations: Such Evidences?. Biomed Res. Int..

[17] Tecco S., Baldini A., Nakaš E., Primozic J. (2018). Orthodontics in Growing Patients: Clinical/Biological Evidence and Technological Advancement 2018. Biomed Res. Int..

[18] Silvestrini Biavati A., Tecco S., Migliorati M., Festa F., Marzo G., Gherlone E., Tetè S. (2011). Three-dimensional tomographic mapping related to primary stability and structural miniscrew characteristics. Orthod. Craniofac. Res..

[19] Tecco S., Farronato G., Salini V., Di Meo S., Filippi M.R., Festa F., D’Attilio M. (2005). Evaluation of cervical spine posture after functional therapy with FR-2: A longitudinal study. Cranio.

[20] Tecco S., Caputi S., Festa F. (2007). Evaluation of cervical posture following palatal expansion: A 12-month follow-up controlled study. Eur. J. Orthod..

[21] Tecco S., Mummolo S., Marchetti E., Tetè S., Campanella V., Gatto R., Gallusi G., Tagliabue A., Marzo G. (2011). SEMG activity of masticatory, neck, and trunk muscles during the treatment of scoliosis with functional braces. A longitudinal controlled study. J. Electromyogr. Kinesiol..

[22] Ciuffolo F., Manzoli L., Ferritto A.L., Tecco S., D’Attilio M., Festa F. (2005). Surface electromyographic response of the neck muscles to maximal voluntary clenching of the teeth. J. Oral. Rehabil..

[23] Giuca M.R., Pasini M., Tecco S., Marchetti E., Giannotti L., Marzo G. (2012). Skeletal maturation in obese patients. Am. J. Orthod. Dentofac. Orthop..

[24] Quinzi V., Scibetta E.T., Marchetti E., Mummolo S., Giannì A.B., Romano M., Beltramini G., Marzo G. (2019). Analyze my face. J. Biol. Regul. Homeost. Agents..

[25] Mummolo S., Marchetti E., Giuca M.R., Gallusi G., Tecco S., Gatto R., Marzo G. (2013). In-office bacteria test for a microbial monitoring during the conventional and self-ligating orthodontic treatment. Head Face Med..

[26] Marure P.S., Patil R.U., Reddy S., Prakash A., Kshetrimayum N., Shukla R. (2011). The effectiveness of pendulum, K-loop, and distal jet distalization techniques in growing children and its effects on anchor unit: A comparative study. J. Indian Soc. Pedod. Prev. Dent..

[27] Ghosh J., Nanda R.S. (1996). Evaluation of an intraoral maxillary molar distalization technique. Am. J. Orthod. Dentofacial Orthop..

[28] Byloff F.K., Darendeliler M.A., Clar E., Darendeliler A. (1997). Distal molar movement using the pendulum appliance. Part 2: The effects of maxillary molar root uprighting bends. Angle Orthod..

[29] Bussick T.J., McNamara J.A. (2000). Dentoalveolar and skeletal changes associated with the pendulum appliance. Am. J. Orthod. Dentofacial Orthop..

[30] Chaqués-Asensi J., Kalra V. (2001). Effects of the pendulum appliance on the dentofacial complex. J. Clin. Orthod..

[31] Fuziy A., Rodrigues de Almeida R., Janson G., Angelieri F., Pinzan A. (2006). Sagittal, vertical, and transverse changes consequent to maxillary molar distalization with the pendulum appliance. Am. J. Orthod. Dentofacial Orthop..

[32] Kalra V. (1995). The K-loop molar distalizing appliance. J. Clin. Orthod..

[33] Ngantung V., Nanda R.S., Bowman S.J. (2001). Posttreatment evaluation of the distal jet appliance. Am. J. Orthod. Dentofacial Orthop..

[34] Bolla E., Muratore F., Carano A., Bowman S.J. (2002). Evaluation of maxillary molar distalization with the distal jet: A comparison with other contemporary methods. Angle Orthod..

[35] Chiu P.P., McNamara J.A., Franchi L. (2005). A comparison of two intraoral molar distalization appliances: Distal jet versus pendulum. Am. J. Orthod. Dentofacial Orthop..

[36] Ravera S., Castroflorio T., Garino F., Daher S., Cugliari G., Deregibus A. (2016). Maxillary molar distalization with aligners in adult patients: A multicenter retrospective study. Prog. Orthod..

[37] Yin K., Han E., Guo J., Yasumura T., Grauer D., Sameshima G. (2019). Evaluating the treatment effectiveness and efficiency of Carriere Distalizer: A cephalometric and study model comparison of Class II appliances. Prog. Orthod..

[38] Bowman A.C., Saltaji H., Flores-Mir C., Preston B., Tabbaa S. (2013). Patient experiences with the Forsus Fatigue Resistant Device. Angle Orthod..

[39] Sandifer C.L., English J.D., Colville C.D., Gallerano R.L., Akyalcin S. (2014). Treatment effects of the Carrière distalizer using lingual arch and full fixed appliances. J. World Fed. Orthod..

[40] Fontana M., Cozzani M., Mutinelli S., Spena R., Caprioglio A. (2015). Maxillary molar distalization therapy in adult patients: A multicentre study. Orthod. Craniofac. Res..

[41] Caruso S., Nota A., Ehsani S., Maddalone E., Ojima K., Tecco S. (2019). Impact of molar teeth distalization with clear aligners on occlusal vertical dimension: A retrospective study. BMC Oral Health.

[42] Hamilton C.F., Saltaji H., Preston C.B., Flores-Mir C., Tabbaa S. (2013). Adolescent patients’ experience with the Carriere distalizer appliance. Eur. J. Paediatr. Dent..

